# Prevalence of mutations in BRCA and MMR genes in patients affected with hereditary endometrial cancer

**DOI:** 10.1007/s12032-021-01454-5

**Published:** 2021-01-23

**Authors:** Maria Teresa Vietri, Giovanna D’Elia, Gemma Caliendo, Amelia Casamassimi, Alessandro Federico, Luana Passariello, Michele Cioffi, Anna Maria Molinari

**Affiliations:** 1 Department of Precision Medicine, University of Campania “Luigi Vanvitelli”, 80138 Naples, Italy; 2 U.O.C. Clinical and Molecular Pathology, A.O.U. University of Campania “Luigi Vanvitelli”, 80138 Naples, Italy; 3 U.O.C. Hepato-Gastroenterology, A.O.U. University of Campania “Luigi Vanvitelli”, 80138 Naples, Italy

**Keywords:** Endometrial cancer, Lynch syndrome, Hereditary breast and ovarian cancer syndrome, MMR genes, BRCA genes

## Abstract

Endometrial cancer (EC) is the fifth most common cancer in women from developed countries, accounting for 4.8% of new cases and 2.1% of deaths. The genetic basis for the familial risk of endometrial cancer has not been completely defined. Mostly, hereditary EC is part of two syndromes as Lynch syndrome (LS) and Hereditary Breast and Ovarian Cancer syndrome (HBOC). LS is the prototypical hereditary cancer syndrome in EC and accounts for 2–6% of all endometrial cancers. This disease is caused by autosomal dominant mutations in DNA mismatch repair (MMR) genes. Patients carrying a germline mutation in one of the MMR genes have a cumulative lifetime risk to develop EC of 20–70%. HBOC is an autosomal dominantly inherited disease, which mostly predisposes to breast and ovarian cancers, but it can be also associated with other malignancies. HBOC results from germline mutations in *BRCA1/2* genes. The aim of this study was to determine the mutational status of a cohort of 40 EC patients, 19 belonging to families with LS and 21 to HBOC. Mutation analysis of *MLH1*, *MSH2*, *BRCA1* and *BRCA2* genes showed pathogenic variants in 17/40 (42.5%) patients. Out of 19 patients belonging to LS families, 8 (42.1%) showed a pathogenic variant. Out of 21 patients belonging to HBOC families, 9 (42.8%) showed a pathogenic variant. 1/21 (4.8%) patient report 1 variant of unknown significance (UV), c.599 C > T (p.T200I), in *BRCA2*. Moreover, in 1/21 (4.8%) patient we identified a novel missense variant in *BRCA2*, c.9541A > T (p.Met3181Leu). Mutational analysis was extended to family members, both healthy and cancer affected, of mutated patients; all the tested relatives affected with cancer displayed the pathogenic variant. Our data suggest that patients with hereditary EC have a high percentage of mutations in the LS and HBOC main susceptibility genes; therefore, the surveillance for EC, already indicated in LS patients, should also be recommended for patients with HBOC.

**Supplementary information:**

The online version of this article (10.1007/s12032-021-01454-5) contains supplementary material, which is available to authorized users.

## Introduction

Endometrial cancer (EC) is the fifth most common cancer in women from developed countries, accounting for 4.8% of new cases and 2.1% of deaths. The highest incidence rates were estimated to be 19.1 and 15.6 per 100,000 in North America and Western Europe respectively [1]. EC is divided into two histologic categories, which differ in incidence and prognosis. Type I tumors comprise approximately 80% of endometrial carcinomas and include low grade [1, 2] tumors of endometrioid histology with a relatively good prognosis. Type II tumors account for approximately 20% of cases and include high grade (grade 3) endometrioid tumors as well as tumors of non-endometrioid histology: serous, clear cell, and other rare subtypes with a relatively poor prognosis [2].

In EC susceptibility, non-genetic risk factors include age and exposure to exogenous estrogens, or endogenous estrogens associated with nulliparity, early age at menarche, late-onset menopause and obesity. Besides, a role is also played by genetic factors, as a family history of EC is associated with a ~ 2–threefold increase risk [3].

The genetic basis for the familial risk of endometrial cancer has not been completely defined. Several mutations of specific genes are the cause of cancer susceptibility syndromes with an elevated risk for endometrial cancer; however, these mutations explain only a fraction of the EC cases [4].

Hereditary EC is part of three syndromes, Lynch syndrome (LS), Hereditary Breast and Ovarian Cancer syndrome (HBOC) and Cowden syndrome.

LS is the prototypical hereditary cancer syndrome in EC and accounts for 2–6% of all endometrial cancers. This disease is caused by autosomal dominant mutations in DNA mismatch repair (MMR) genes [5]. MMR genes mainly include *MLH1*, *MSH2*, *MSH6*, and *PMS2*. An estimated 70%–90% of LS is attributable to deleterious mutations in *MLH1* and *MSH2*, with the remaining 10%–30% distributed between *MSH6* and *PMS2* [6]. Patients who carry a germline mutation in one of the MMR genes have a cumulative lifetime risk to develop EC of 20–70% [7], particularly women with *MSH2* or *MLH1* mutations have a 40% to 50% risk of EC manifestation. MMR gene mutations increase the incidence not only of colorectal cancer, but also of ovarian, breast, gastric, pancreatic, biliary tract, small bowel, and urothelial cancers in individuals who harbor deleterious mutations and among family members [8].

The HBOC syndrome is an autosomal dominantly inherited disease that predisposes mostly to breast and ovarian cancers; it is characterized by a young age of onset, more than one synchronous or metachronous tumor, bilateral breast cancer and a family history of first- and second-degree relatives with similar cancers. Largely, HBOC syndrome results from germline mutations in breast cancer genes *BRCA1* or *BRCA2*. In HBOC patients, an increased risk of other neoplasms including prostate cancer, pancreatic cancer, gastric cancer and cutaneous malignant melanoma, has been reported, especially in individuals with germline *BRCA2* mutations [9]. Whether EC should be considered part of HBOC syndrome is still under debate. A number of studies showed an increased risk to develop EC, especially in *BRCA1* mutations carriers, with the highest observed risk for an aggressive EC subtype, the serous-like [10].

Cowden syndrome is a rare condition resulting from a mutation in the phosphatase and tensin homolog (PTEN) tumor suppressor gene. This syndrome is characterized by hamartomatous tumors in multiple organ systems and includes an increased risk of EC [4]. The lifetime risk of EC in women with Cowden syndrome is estimated to be 10–28% [11].

The aim of this study was to determine the mutational status in a cohort of EC patients belonging to Italian families with LS and HBOC and to extend the genetic analysis to their relatives.

## Materials and methods

### Patients

This study was carried out in accordance with the World Medical Association Helsinki Declaration, adopted in 1964 and amended in 1975, 1983, 1989, 1996 and 2000 (World Medical Association, 1998). Informed consents were obtained from all subjects, and the study was approved and conducted according to the ethical guidelines of the University of Campania “Luigi Vanvitelli” (n.469–23/07/2019).

In this study, we enrolled 40 patients with EC (range age 20–80 years). Nineteen patients were from LS families; particularly, 16 affected with EC, 2 with EC and colon cancer (CC) and 1 with EC, CC and gastric cancer. Twenty-one patients belonged to HBOC families, 15 affected with EC and 6 with EC and breast cancer. None of the analyzed patients belonged to families with Cowden syndrome.

The main clinical and histopathological data were acquired by genetic counselling. The patients were selected according to the criteria for LS and HBOC.

The Amsterdam and the Bethesda criteria can be used to identify individuals at risk for LS. The Amsterdam criteria include at least three relatives with HNPCC-related malignancies (colon, endometrium, small bowel, renal pelvis, or ureter), one affected person is a first-degree relative of the other two affected family members, at least two successive generations are affected, one affected person is diagnosed at younger than age 50, familial adenomatous polyposis is excluded, and tumors are verified by a pathologist [12]. The Bethesda guidelines include CC diagnosed in a patient before age 50; the presence of synchronous, metachronous colorectal, or other HNPCC associated tumors (colorectal, endometrial, stomach, ovarian, pancreatic, ureter, renal pelvis, biliary tract, brain, or small bowel); CC with MSI-high-like histology in a patient younger than 60 years; CC in a patient with one or more first-degree relatives with an HNPCC-related tumor, with one of the cancers being diagnosed before age 50; and CC in a patient with two or more first-degree relatives with HNPCC-related tumors, regardless of age [13].

Criteria to identify individuals with HBOC syndrome include women with synchronous or metachronous breast and ovarian cancer; breast cancer ≤ 40 years; bilateral breast cancer (the first diagnosed ≤ 50 years); triple-negative breast cancer ≤ 60 years; high-grade epithelial non-mucinous ovarian cancer (or fallopian tube or primary peritoneal cancer); ancestry with founder mutations; BRCA somatic mutation detected in any tumor type with a allele frequency > 30% (if it is known); metastatic HER2-negative breast cancer patients eligible to consider PARP inhibitor therapy; 2 or more first degree relatives with any combination of the following high-risk features; bilateral breast cancer + another breast cancer < 60 years; breast cancer < 50 years and prostate or pancreatic cancer < 60 years; male breast cancer; breast and ovarian cancer; two cases of breast cancer diagnosed before age 50 years; 3 or more direct relatives with breast cancer (at least one premenopausal) and/or ovarian cancer and/or pancreatic cancer or high Gleason (≥ 7) prostate cancer [14]. Peripheral blood samples were collected in two test tubes from all patients. Mutational analysis was extended to family members of mutated patients.

### Mutation analysis

The extraction of genomic DNA from peripheral blood lymphocytes was performed using Wizard Genomic DNA purification kit (Promega Corporation, Madison, WI, USA).

Mutational analysis of exons of *MLH1*, *MSH2*, *BRCA1*, *BRCA2* and adjacent intronic regions was performed with NGS, as previously described [15, 16]. The presence of the pathogenic variant was confirmed on the other blood sample by Sanger sequencing, as previously described [17]. Mutational analysis in the family members of mutated patients was conducted by Sanger sequencing.

### In Silico analysis

To evaluate a potential effect of the variant found, the integrated software Alamut V.2.11 (January 2018) (Interactive Biosoftware, Rouen, France. Available at http://www.interactive-biosoftware.com). This software included three prediction algorithms viz. Align GVGD, SIFT and MutationTaster. The genomic sequence spanning the individual mutations and nearby exons was submitted according to the guidelines of each program and default settings were used in all the predictions.

## Results

Mutation analysis of *MLH1*, *MSH2*, *BRCA1* and *BRCA2* genes performed on 40 patients with hereditary EC showed pathogenic variants in 17/40 (42.5%) patients.

### LS patients

**Fig. 1:**
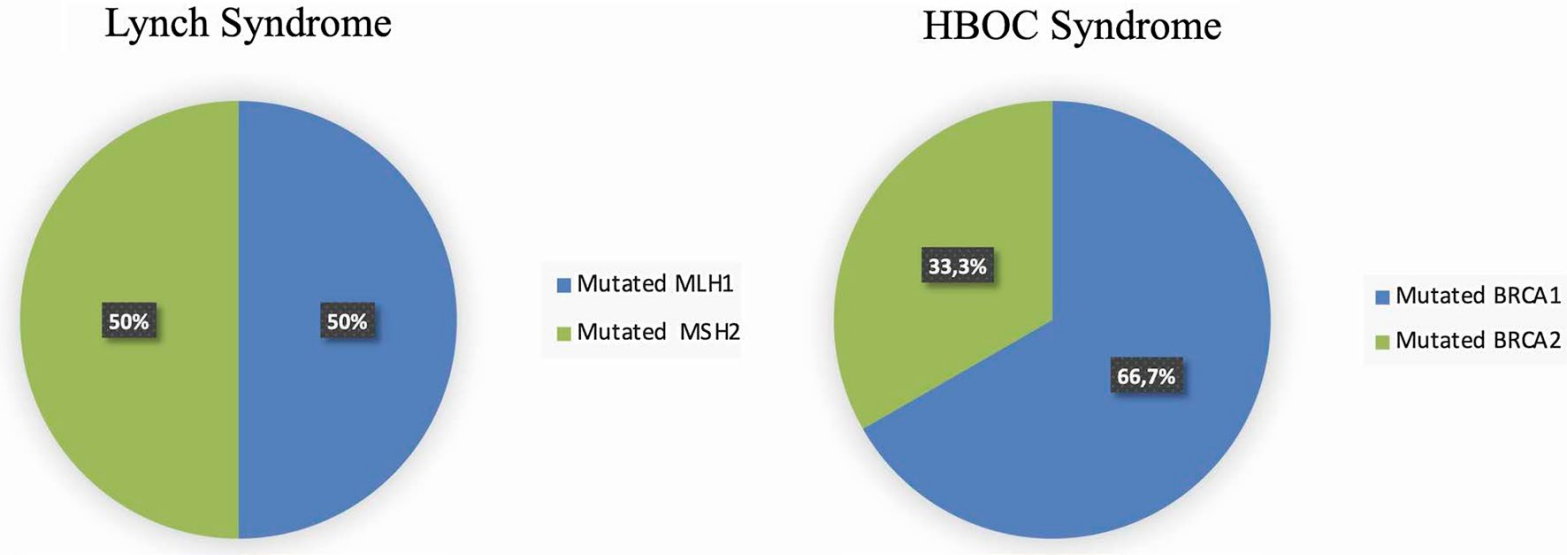
The frequency of pathogenic variants found in *BRCA1*, *BRCA2*, *MLH1* and *MSH2* genes in EC patients

Out of 19 patients belonging to LS families, 8 (42.1%) showed a pathogenic variant, 4 (50%) in *MLH1* and 4 (50%) in *MSH2* genes. The graph in Fig. 1 illustrates the percentage of pathogenic variants found in the analyzed genes.

The clinical characteristic of mutated patients with LS and the type of pathogenic variant identified are detailed in Table 1. In the group of analyzed patients, 50% of patients with *MLH1* pathogenic variant and 25% of patients with *MSH2* pathogenic variant reported a personal history of LS-related cancers. The mean age of EC onset was 35.6 years, 31 years for *MLH1* mutated patients and 40.2 years for *MSH2* mutated patients.

**Table 1:** Patients affected with hereditary EC carrying *MLH1* or *MSH2* pathogenic variants, belonging to LS families

Proband	Diagnosis	Age at sampling	Age at diagnosis	MLH1 pathogenic variants	MSH2 pathogenic variants	Molecular Consequence
1	EC	29	27	c.229 T > C (p.Cys77Arg)		Missense
2	EC	47	38	c.229 T > C (p.Cys77Arg)		Missense
3	EC–CC	59	39 – 58	c.683dupT (p.Ile229Aspfs)		Frameshift
4	EC–CC	22	20 – 21	c.954delC (p.His318Glnfs)		Frameshift
5	EC	33	31		c.942 + 2 T > A (IVS5 + 2 T > A)	Splice
6	EC- GC–CC	55	54 -52–48		c.942 + 3A > T (IVS5 + 3A > T)	Splice
7	EC	45	36		c.1147C > T (p.Arg383Ter)	Nonsense
8	EC	42	40		c.1213delT(p.Tyr405Thrfs)	Frameshift

*EC* Endometrial cancer, *CC* Colon cancer, *GC* Gastric cancer

The pedigrees of the 8 LS mutated patients are reported in Supplementary Figures S1-S8. Out of the 8 probands, 3 were affected by two or more tumors, particularly the probands of families 3 and 4 were affected by EC and CC, the proband of family 6 was affected by EC, CC and gastric cancer. Moreover, we observed the presence of tumors also in family members. Specifically, in family 1, one member was affected by two tumors; in family 2, three family members reported two or more tumors; one and two family members were affected with two tumors in families 6 and 7 respectively.

Mutational analysis was extended to 25 family members, both healthy and cancer affected, of 6 LS mutated patients, while for the remaining two LS patients (family 5 and 6) the analysis of family members could not be performed. Out of the 25 family members investigated, 7 were not mutated. In Table 2, the probands and their mutated relatives are reported. All the tested relatives affected with cancer displayed the pathogenic variant.

**Table 2:** Characteristics of LS family members carrying a MMR mutation

Family N°	Mutation	Gene	Family members	Diagnosis	Age
1	c.229 T > C (p.Cys77Arg)	MLH1	Proband	EC	29 (27)
			Cousin	Colon cancer	28 (28)
2	c.229 T > C (p.Cys77Arg)	MLH1	Proband	EC	47 (38)
			Daughter	Unaffected	26
			Son	Unaffected	19
			Brother	Parathyroid cancer	44 (37)
			Cousin	Ovarian cancer	38 (36)
4	c.954delC (p.His318Glnfs)	MLH1	Proband	EC Colon cancer	22 (20) (21)
			Cousin	Breast cancer	48 (47)
			Cousin	Colon cancer	50 (45)
7	c.1147C > T (p.Arg383Ter)	MSH2	Proband	EC	45 (36)
			Sister	Unaffected	38
			Cousin	Unaffected	35
			Cousin	Unaffected	24
			Cousin	Colon cancer	51 (49)
			Aunt	Colon cancer Bladder cancer	56 (47) (52)
			Aunt	Ovarian cancer	59 (47)
8	c.1213delT (p.Tyr405Thrfs)	MSH2	Proband	EC	42 (40)
			Mater	EC	75 (56)
			Cousin	Unaffected	50
			Aunt	EC	66 (60)
			Aunt	EC	77 (52)
			Aunt	EC	66 (57)

The ages at diagnosis are indicated in brackets

**Fig. 2:**
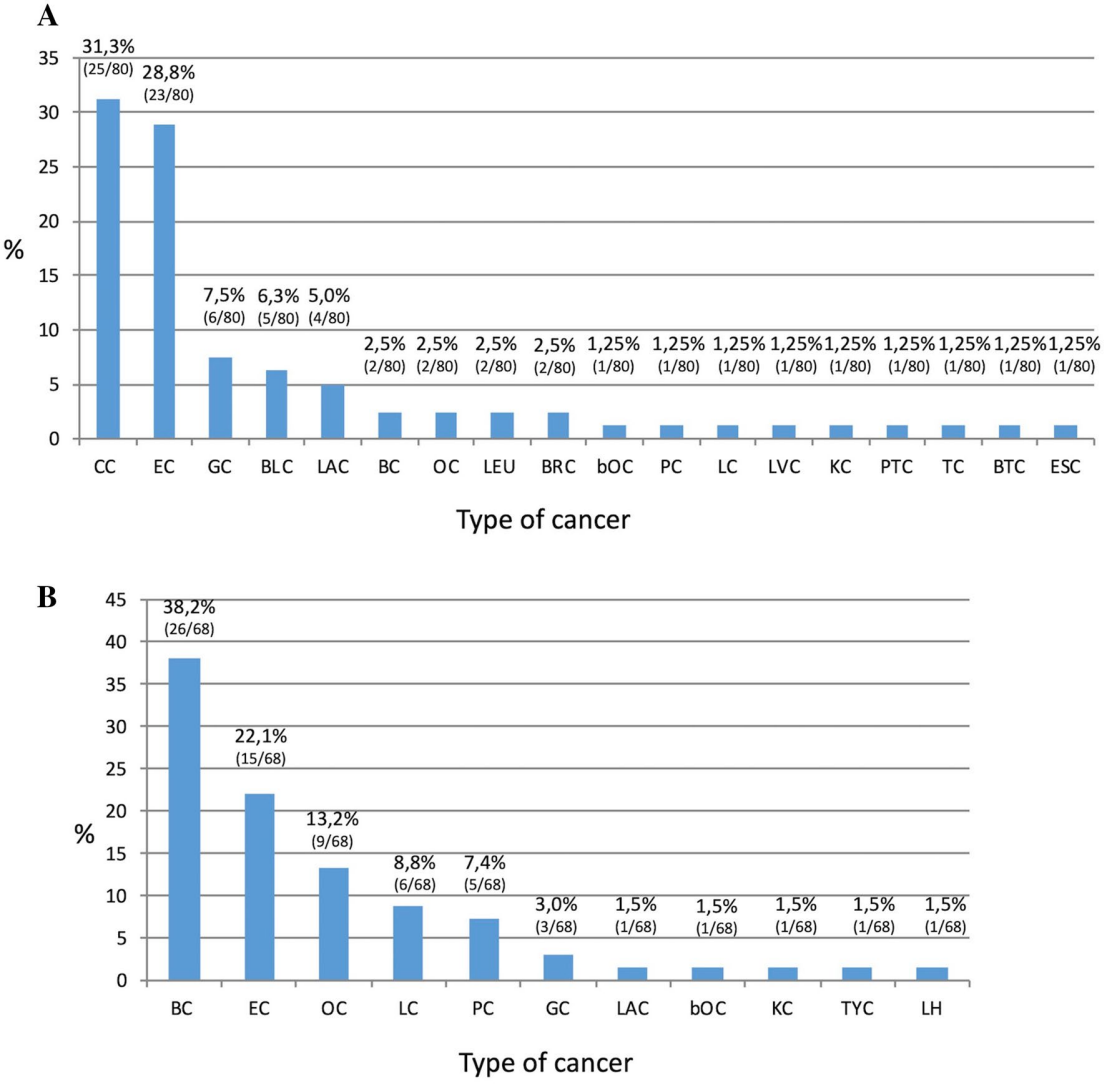
Number of cancer types occurring in families of LS mutated patients (**a**) or HBOC mutated patients (**b**). *CC* Colon cancer, *EC* Endometrial cancer, *GC* Gastric cancer, *BLC* Bladder cancer, *LAC* Laryngeal cancer, *BC* Breast cancer, *OC* Ovarian cancer, *LEU* Leukemia, *BRC* Brain cancer, *bOC* Bilateral Ovarian cancer, *PC* Prostate cancer, *LC* Lung cancer, *LVC* Liver cancer, *KC* Kidney cancer, *PTC* Parathyroid cancer, *TC* Testicular cancer, *BTC* Biliary tract cancer, *ESC* Esophageal cancer, *TYC* Thyroid cancer, *LH* Hodgkin lymphoma

Figure 2a shows EC and other cancer cases in the LS families. The most frequent tumor was CC (31.3%), followed by EC (28.8%). To a lesser extent, other tumors could be observed, including gastric cancer (7.5%), bladder cancer (6.3%), laryngeal cancer (5.0%) and in low percentages BC, OC and prostate cancer.

The mean age of EC onset in families with LS was 42 years.

### HBOC patients

Out of 21 patients belonging to HBOC families, 9 (42.8%) showed a pathogenic variant, 6 (66.7%) in *BRCA1* and 3 (33.3%) in *BRCA2* genes. Figure 1 illustrates the percentage of pathogenic variants found in analyzed genes.

The clinical characteristic of mutated patients with HBOC and the type of pathogenic variant identified are described in Table 3. Specifically, 33.3% of mutated patients reported a personal history of other cancer. The mean age of EC onset was 51.5 years, 49.5 years for patients mutated in *BRCA1* and 55.7 years for patients mutated in *BRCA2*.

**Table 3:** Patients affected with hereditary EC carrying *BRCA1* or *BRCA2* pathogenic variants, belonging to HBOC families

Proband	Diagnosis	Age at sampling	Age at diagnosis	BRCA1 pathogenic variants	BRCA2 pathogenic variants	Molecular Consequence
9	EC	72	71	c.181 T > G (p.Cys61Gly)		Missense
10	EC	52	33	c.213-11 T > G (IVS5-11 T > G)		Splice
11	EC	50	48	c.547 + 2 T > A (IVS8 + 2 T > A)		Splice
12	EC—BC	59	54 – 56	c.2952delT (p.Ile986Serfs)		Frameshift
13	EC	68	49	c.4484G > T (p.Arg1495Met)		Missense
14	EC—BC	43	42 – 42	c.5266dupC (p.Gln1756Profs)		Frameshift
15	EC—BC	70	62 – 61		NS1742del (p.N1742_S1743del)	Frameshift
16	EC	53	50		c.4131_4132insTGAGGA (p.Thr1378Ter)	Frameshift
17	EC	80	55		c.8954-1_8955delGTTinsAA (IVS22-1del3insAA)	Frameshift

*EC* Endometrial cancer, *BC* Breast cancer

Moreover, 1/21 (4.8%) HBOC patient showed a variant of unknown significance (UV), c.599 C > T (p.T200I), in *BRCA2* gene. In 1/21 (4.8%) patient we identified a novel missense variant in the exon 26 of *BRCA2*, c.9541A > T (p.Met3181Leu).

To evaluate the potential pathogenetic effect, we conducted the *in-silico* analysis using three different prediction programs. Overall, in silico analysis predicted c.9541A > T (p.Met3181Leu) as tolerated in three classifier algorithms.

The pedigrees of 9 mutated patients with HBOC are shown in Supplementary figures S9-S17.

Out of the 9 probands, 3 were affected by two tumors, particularly the probands of families 12, 14 and 15 were affected by EC and BC. Moreover, we observed the presence of different tumors also in family members, but no family member was affected by two or more tumors.

Mutational analysis was extended to 23 family members, both healthy and cancer affected, of 7 HBOC mutated patients. For 2 HBOC mutated probands (family 11 and 14) we could not apply the genetic test to the relatives.

Out of the 23 family members investigated, 11 were not mutated. Table 4 reports the probands and their mutated relatives. As for LS cases, also the cancer affected family members of HBOC probands reported the pathogenic variant.

**Table 4:** Characteristics of HBOC family members carrying a BRCA mutation

Family N°	Mutation	Gene	Family members	Diagnosis	Age
9	c.181 T > G (p.Cys61Gly)	BRCA1	Proband	EC	72 (71)
			Sister	Breast cancer	64 (42)
10	c.213-11 T > G (IVS5-11 T > G)	BRCA1	Proband	EC	52 (33)
			Brother	Unaffected	59
			Daughter	Unaffected	24
13	c.4484G > T (p.Arg1495Met)	BRCA1	Proband	EC	68 (49)
			Daughter	Ovarian cancer	48 (48)
15	NS1742del (p.N1742_S1743del)	BRCA2	Proband	EC Breast cancer	70 (62) (61)
			Nephew	Breast cancer	52 (49)
16	c.4131_4132insTGAGGA (p.Thr1378Ter)	BRCA2	Proband	EC	53 (50)
			Cousin	Ovarian cancer	61 (61)
			Daughter	Unaffected	40
17	c.8954-1_8955delGTTinsAA (IVS22-1del3insAA)	BRCA2	Proband	EC	80 (55)
			Nephew	Prostate cancer	66 (59)
			Nephew	Thyroid cancer	70 (65)
			Nephew	Gastric cancer	74 (54)
			Great granddaughter	Ovarian cancer	44 (44)
			Great granddaughter	Unaffected	44

The ages at diagnosis are indicated in brackets

Figure 2b shows EC and other cancer cases in the HBOC families. The most frequent tumor was BC (38.2%), followed by EC (22.1%) and OC (13.2%). To a lesser extent, other tumors were observed including prostate cancer (7.4%), gastric cancer (3.0%) and bilateral ovarian cancer (1.5%).

The mean age of EC onset in HBOC families was 54.5 years.

## Discussion

In this study, we analyzed Hereditary EC cases as part of two syndromes, LS and HBOC. Specifically, we report the results of mutation analysis of the main susceptibility genes to such syndromes, *MLH1*, *MSH2*, *BRCA1* and *BRCA2*, performed on patients affected with EC and belonging to LS and HBOC families.

Approximately 5% of all ECs are caused by mutations in MMR genes. The lifetime risk of developing EC is approximately 2.9% in the general population compared with the 21‐54% lifetime risk for mutated women with LS, depending on the mutation type [18].

*MSH2* is the most frequently mutated gene in women with EC associated with LS and it is reported in 50–66% of EC cases with a mutation. Mutations in *MLH1* occur in 24–40%, and in *MSH6* in 10–13%, of cases [19].

Our study reports a higher mutation rate in the MMR genes (42.1%), compared to other studies (18–22.7%) [16, 20]. Particularly, in our cohort of patients, we found no differences in the percentage of pathogenic variants in the analyzed genes, as we revealed a percentage of 50% in both *MLH1* and *MSH2*. A previous study on EC patients found variants most frequently in the *MSH2* gene (43%) followed by *MSH6* (24%) and *MLH1* (22%) [20]. Therefore, the percentage of mutations in MMR genes changes in the various populations. The mutated LS patients developed EC at mean age of 35.6 years, particularly patients carrying *MLH1* pathogenic variants developed EC at a younger age (mean age = 31) than did *MSH2* pathogenic variant carriers (mean age = 40.2). The mean age of EC onset in families with LS was 42 years. This finding confirms that in LS patients EC tends to occur at a younger age than in sporadic cases. In the previous report, patients carrying *MLH1* variants developed EC at a younger age (mean age = 46.4) than did *MSH2* variant carriers (mean age = 51.9) [20]. Thus, in our patients the observed EC onset was even earlier.

In EC patients with MMR mutation, a personal history of LS-related cancers was described in 50% of *MLH1* mutation carriers and in 45% of *MSH2* mutation carriers [20]. In our cohort, we have found a personal history of LS-related cancers in 50% of patients with *MLH1* pathogenic variants and in 25% of patients with *MSH2* pathogenic variants. Patients carrying *MLH1* pathogenic variants were affected by EC and CC, the patient with a pathogenic variant in *MSH2* was affected by EC, CC and gastric cancer (GC). Moreover, a personal history of LS-related cancers was also observed in 10.3% of LS family members (Supplementary Figures S1-S8).

Besides, in families of the mutated patients not only other EC cases occur, but also other LS-related cancers, as shown in Fig. 2a. In addition to EC, CC is the most frequent (31.3% of LS families) confirming that CC risk is high in LS patients and varies according to the involved gene.

GC occurs in 7.5% of our cases. This cancer type is reported in approximately 5–13% of LS individuals. Risks are higher in *MLH1* and *MSH2* than other mutation carriers, and higher in males than in females [21].

Bladder cancer is present in 6.3% of patients. Recent data suggest a two- to four-fold elevated risk of bladder cancer, with the highest risk occurring in men with *MSH2* mutations [22].

The relationship between breast cancer (BC) and LS remains unresolved. Studies have not consistently demonstrated a higher incidence of BC among individuals with LS than expected [23]. Likewise, in our patients BC occurs in 2.5% of cases. As BC is fairly common in the general population, larger studies are needed to determine whether BC is indeed part of the LS cancer spectrum.

Approximately 2% of ovarian cancers (OC) are due to LS. In the families of our mutated patients OC was observed in 2.5% of cases. Reported lifetime risks for OC in women with LS fall primarily within the range of 3‐20% and appear highest for carriers of *MSH2* mutations, followed by *MSH6* and *MLH1* [18].

Individuals with LS have up to a 3% lifetime risk of developing cancers of the brain [24], which was present in 2.5% of our families.

Prostate cancer has been also associated with LS; several studies have found the lifetime risk for prostate cancer in LS to be increased by two- to five-fold [25]. In our families, prostate cancer was reported by 1.25% of patients. Additional studies are needed to determine whether LS-associated prostate cancers occur at an earlier average age or are more aggressive.

Up to 4% of people with LS develop liver cancer by age of 70 years another rare cancer in the general population [21]. It was diagnosed in 1.25% of our patients.

The spectrum of LS-associated tumors is wide, and several very rare cancers in the general population are seen more frequently in this syndrome. Although the risks for these rare tumors are greatly increased above the general population risks, the absolute risks are low. Additionally, LS-related cancers in family members were more common in *MLH1* (70%) than *MSH2* variant carriers (65%) [20].

In EC patients belonging to HBOC families we found a high mutation rate of 42.8%, with 66.7% in *BRCA1* and 33.3% in *BRCA2*.

Pennington et al. in 2013 sequenced 30 candidate tumor suppressor genes in 151 patients with uterine carcinoma and found the prevalence of germline mutations in *BRCA1* to be 2% (26). Afterwards, four other studies involving only Jewish patients found an increased mutation rate in *BRCA1* between 14 and 27%, which is significantly higher than the 2% [27].

In our study, the percentage of pathogenic variants in these two genes was very high (42.8%); thus, this is the first study with such a high percentage among the few studies correlating EC with pathogenic variants in *BRCA* genes. In addition, the age of EC onset was relatively low, 51.5 years in mutated patients and 54.5 years in HBOC family members.

A personal history of BC was described in 50% and 100% of patients with *BRCA1/2* mutations [27]; particularly, a personal history of BC was found in 16.4% of EC patients [26]. In our study, 6/21 of HBOC patients (28.6%) had a personal history of BC and EC; 3 of them were mutated, suggesting that EC is not only one of the HBOC-related tumors, but patients with BC risk can develop EC other than OC.

Several studies suggest that *BRCA* mutation carriers display an increased risk of papillary serous carcinoma of the endometrium [28]. It has been highlighted that HBOC-associated EC tend to be of serous papillary type, an aggressive histologic subtype [29] that accounts for less than 10% of EC (20) whereas HNPCC-associated EC typically are of endometrial type [30]. This finding was confirmed in other studies focused on the incidence of *BRCA* founder mutations in patients with uterine serous carcinoma. A first report on 22 cases of uterine serous cancer found that 6 patients, accounting for 27%, had a germline mutation in *BRCA1* or *BRCA2* [31]. In other studies, a significantly higher mutation rate in *BRCA1* (11.9%) than in *BRCA2* mutations (1.7%) was found [5, 26, 32]. These results indicated that uterine serous cancer is more commonly associated with mutations in *BRCA1* than *BRCA2*.

Unfortunately, we do not have information on the specific histologic subtype of EC of our patients. These data suggest that in HBOC patients the oncological prevention path should be performed not only for OC onset but also for EC.

In our cohort BC was observed in 38.2% of cases (Fig. 2b), which is a very high percentage; indeed, a family history of BC was found in 29.9% of EC patients [26]. As shown, BC is the most frequent cancer, confirming that the BC risk in *BRCA1* and *BRCA2* mutation carriers is 45–80% [33].

Moreover, *BRCA1* and *BRCA2* mutation carriers have a risk of OC onset of 45–60% and 11–35% respectively; accordingly, in the families of our patients, OC was reported in 13.2% of cases (Fig. 2b).

In a smaller percentage, we also found the presence of other tumors, among them prostate cancer occurred in 7.4% of cases and GC was found in 3% of cases. It has been highlighted that *BRCA1/2* mutation carriers present an increased risk for prostate cancer (3.4-fold in *BRCA1*, 8.6-fold in *BRCA2*) [34]. Moreover, an increased frequency of other malignancies, such as gastro-intestinal tumors, has been reported in families with mutations in the *BRCA2* gene [34].

Moreover, in 1.5% of cases, we found Hodgkin lymphoma, laryngeal, kidney and thyroid cancers that are known to be part of the HBOC (Fig. 2b).

Finally, 1/21 (4.8%) patient reported a variant of unknown significance (UV), c.599 C > T (p.T200I), in *BRCA2* gene. This variant was observed in a family affected with breast and ovarian cancer. Algorithms developed to predict the effect of missense changes on protein structure and function (SIFT, PolyPhen-2, Align-GVGD) all suggest that this variant is likely to be disruptive, but these predictions have not been confirmed by published functional studies. Experimental studies on the effect of this variant on mRNA splicing are contradictory [35, 36]. The available evidence is currently insufficient to determine the role of this variant in disease. Therefore, we classified as a UV.

Furthermore, in 1/21 (4.8%) patient we have identified a novel missense variant in *BRCA2*, c.9541A > T (p.Met3181Leu), which has not been described yet. *In-silico* analysis indicates that this variant may have a benign effect; however, the evaluation of the pathogenetic significance needs to be corroborated by further experimental evidence.

Our data suggest that patients with hereditary EC have a high percentage of mutations in the main susceptibility genes to LS and HBOC. To our knowledge, there are no current published studies that have found hotspot mutations in these genes correlating with EC; therefore, it would be interesting to carry out further studies that evaluate a possible genotype–phenotype correlation.

Moreover, since EC occurs in mutation carriers at an early age, these at-risk individuals should undergo cancer prevention routes not only for the most frequent tumors but also for EC. The screening for EC among LS patients has been recommended by numerous experts; indeed, there is evidence that EC is often a sentinel cancer for women with LS. This implementation of cancer prevention should also be extended also to HBOC patients.

## Supplementary information

Below is the link to the electronic supplementary material.

Supplementary information 1 (PDF 272 kb)

Supplementary information 2 (DOCX 19 kb)

## Data Availability

All data generated or analyzed in the current study are included in this publication and are available on reasonable request.
