# Supplementary material for: Prevalence of mutations in BRCA and MMR genes in patients affected with hereditary endometrial cancer
Source: Med Oncol. 2021 Jan 23;38(2):13. doi: 10.1007/s12032-021-01454-5 (PMC7826304; doi:10.1007/s12032-021-01454-5)
Supplement: Supplementary file 1 — Supplementary information 1 (PDF 272 kb) [file 12032_2021_1454_MOESM1_ESM.pdf]

Family 1

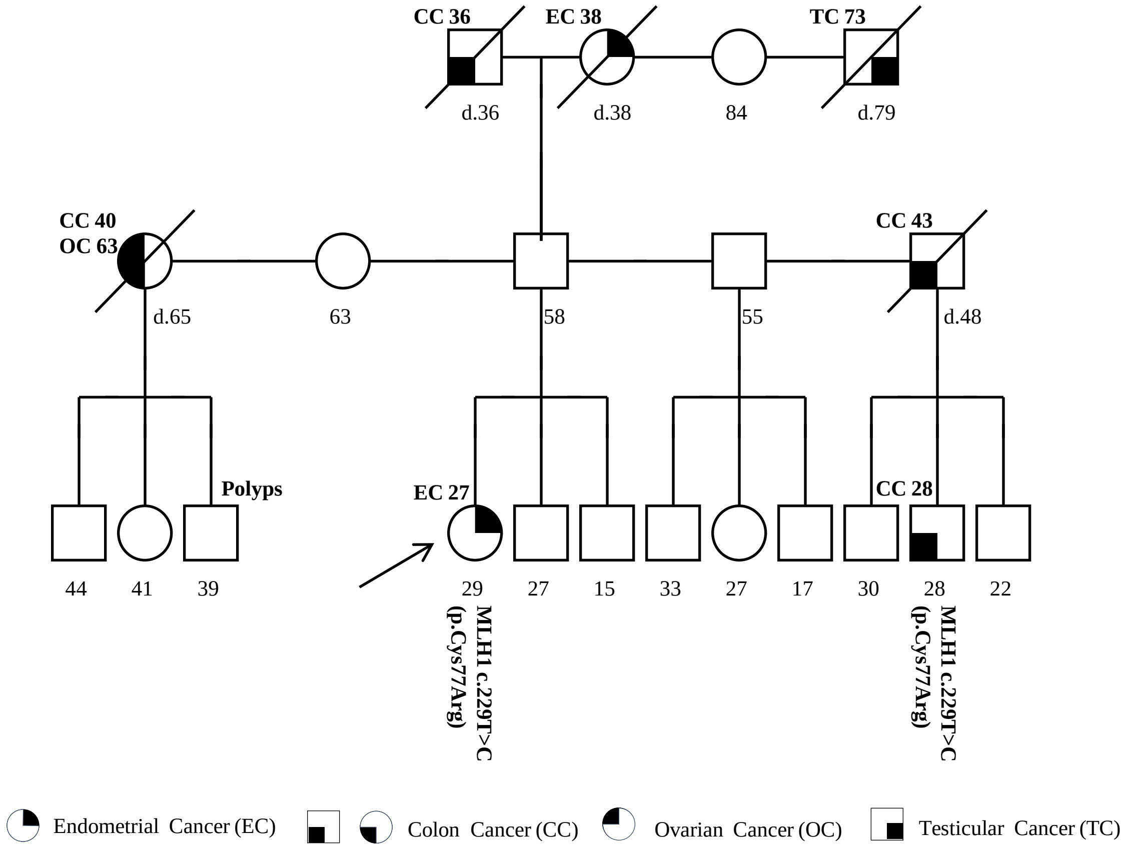
CC 36
EC 38
TC 73
d.36
d.38
84
d.79
CC 40
OC 63
CC 43
d.65
63
58
55
d.48
Polyps
EC 27
CC 28
44
41
39
29
27
15
33
27
17
30
28
22
MLH1 c.229T>C (p.Cys77Arg)
MLH1 c.229T>C (p.Cys77Arg)
Endometrial Cancer (EC)
Colon Cancer (CC)
Ovarian Cancer (OC)
Testicular Cancer (TC)

Family 2

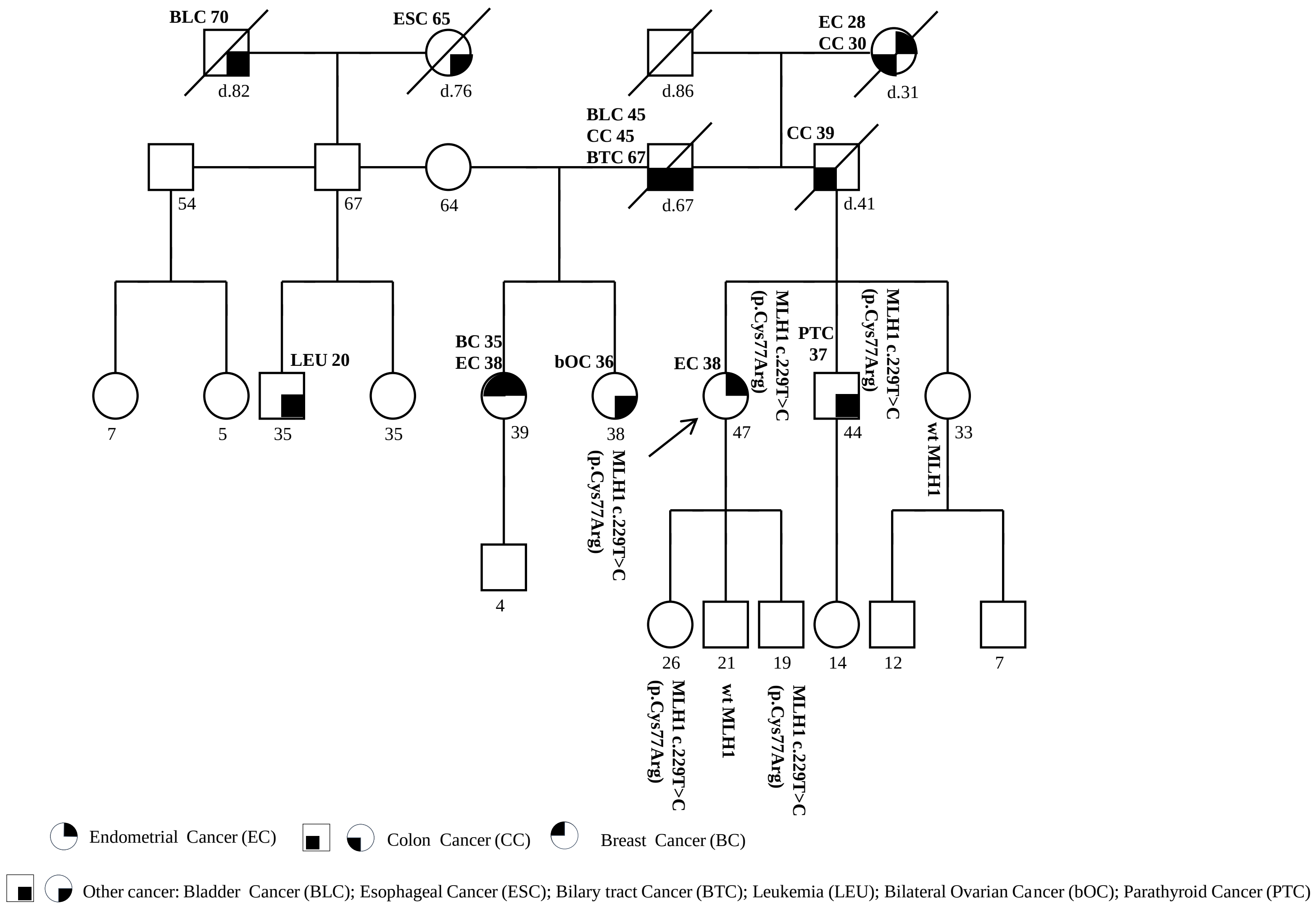
BLC 70
d.82
ESC 65
d.76
d.86
EC 28
CC 30
d.31
54
67
64
BLC 45
CC 45
BTC 67
d.67
CC 39
d.41
7
5
LEU 20
35
35
BC 35
EC 38
39
bOC 36
38
MLH1 c.229T>C (p.Cys77Arg)
EC 38
47
MLH1 c.229T>C (p.Cys77Arg)
PTC 37
44
MLH1 c.229T>C (p.Cys77Arg)
33
wt MLH1
4
26
MLH1 c.229T>C (p.Cys77Arg)
21
wt MLH1
19
MLH1 c.229T>C (p.Cys77Arg)
14
12
7
Endometrial Cancer (EC)
Colon Cancer (CC)
Breast Cancer (BC)
Other cancer: Bladder Cancer (BLC); Esophageal Cancer (ESC); Bilary tract Cancer (BTC); Leukemia (LEU); Bilateral Ovarian Cancer (bOC); Parathyroid Cancer (PTC)

Family 3

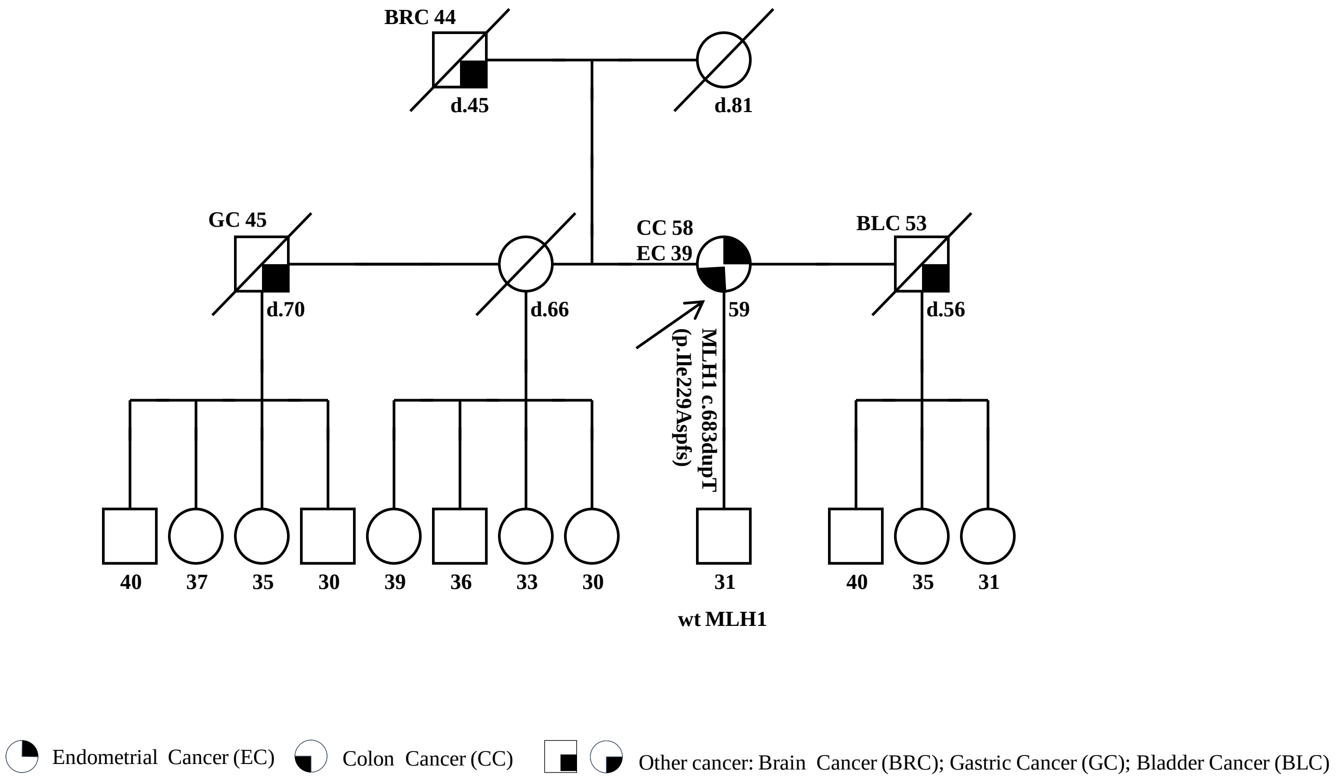

BRC 44
d.45
d.81
GC 45
d.70
d.66
CC 58
EC 39
59
BLC 53
d.56
MLH1 c.683dupT
(p.Ile229Aspfs)
40
37
35
30
39
36
33
30
31
wt MLH1
40
35
31
Endometrial Cancer (EC)
Colon Cancer (CC)
Other cancer: Brain Cancer (BRC); Gastric Cancer (GC); Bladder Cancer (BLC)

Family 4

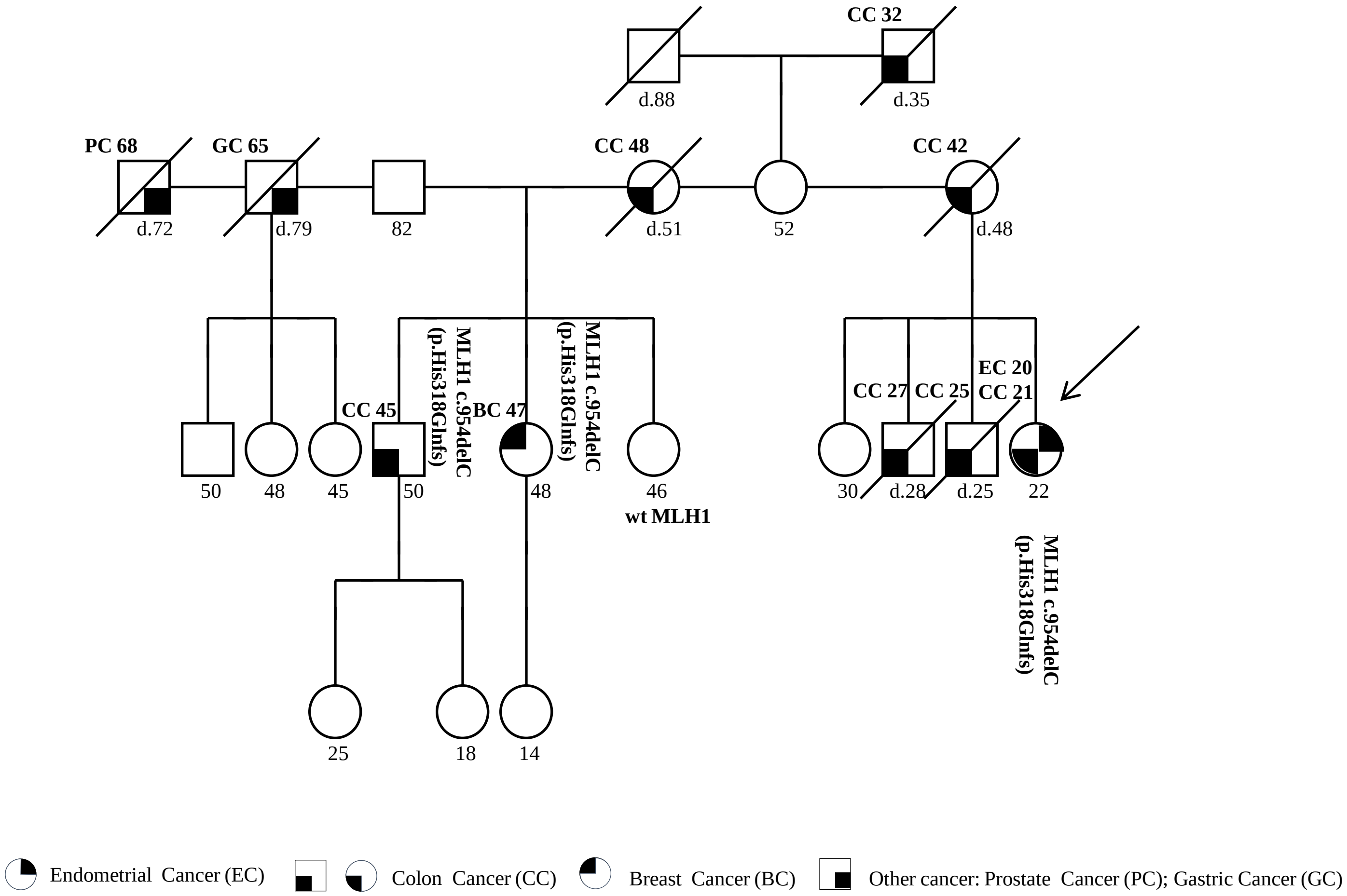
CC 32
d.88
d.35
PC 68
GC 65
CC 48
CC 42
d.72
d.79
82
d.51
52
d.48
CC 45
BC 47
MLH1 c.954delC (p.His318Glnfs)
MLH1 c.954delC (p.His318Glnfs)
CC 27
CC 25
EC 20
CC 21
50
48
45
50
48
46
wt MLH1
30
d.28
d.25
22
MLH1 c.954delC (p.His318Glnfs)
25
18
14
Endometrial Cancer (EC)
Colon Cancer (CC)
Breast Cancer (BC)
Other cancer: Prostate Cancer (PC); Gastric Cancer (GC)

Family 5

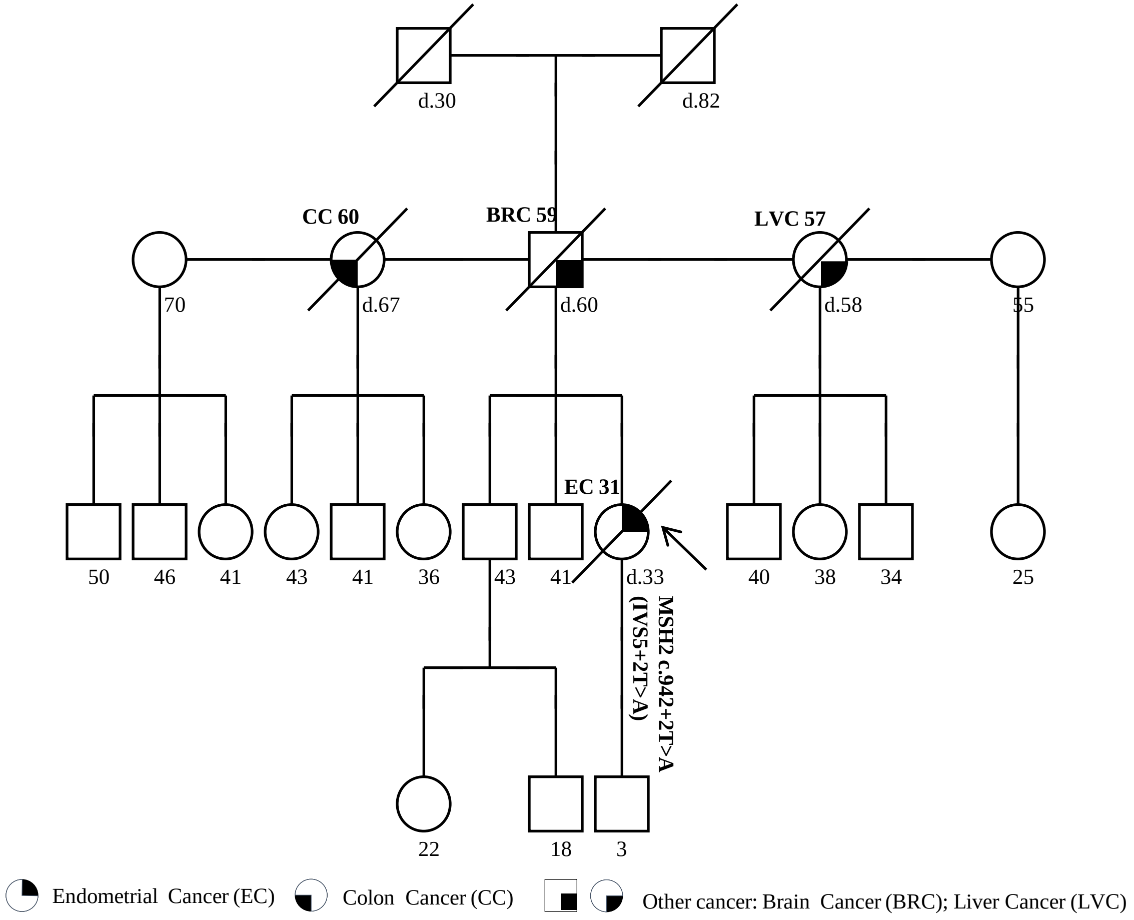
d.30
d.82
CC 60
BRC 59
LVC 57
70
d.67
d.60
d.58
55
EC 31
50
46
41
43
41
36
43
41
d.33
40
38
34
25
MSH2 c.942+2T>A
(IVS5+2T>A)
22
18
3
Endometrial Cancer (EC)
Colon Cancer (CC)
Other cancer: Brain Cancer (BRC); Liver Cancer (LVC)

Family 6

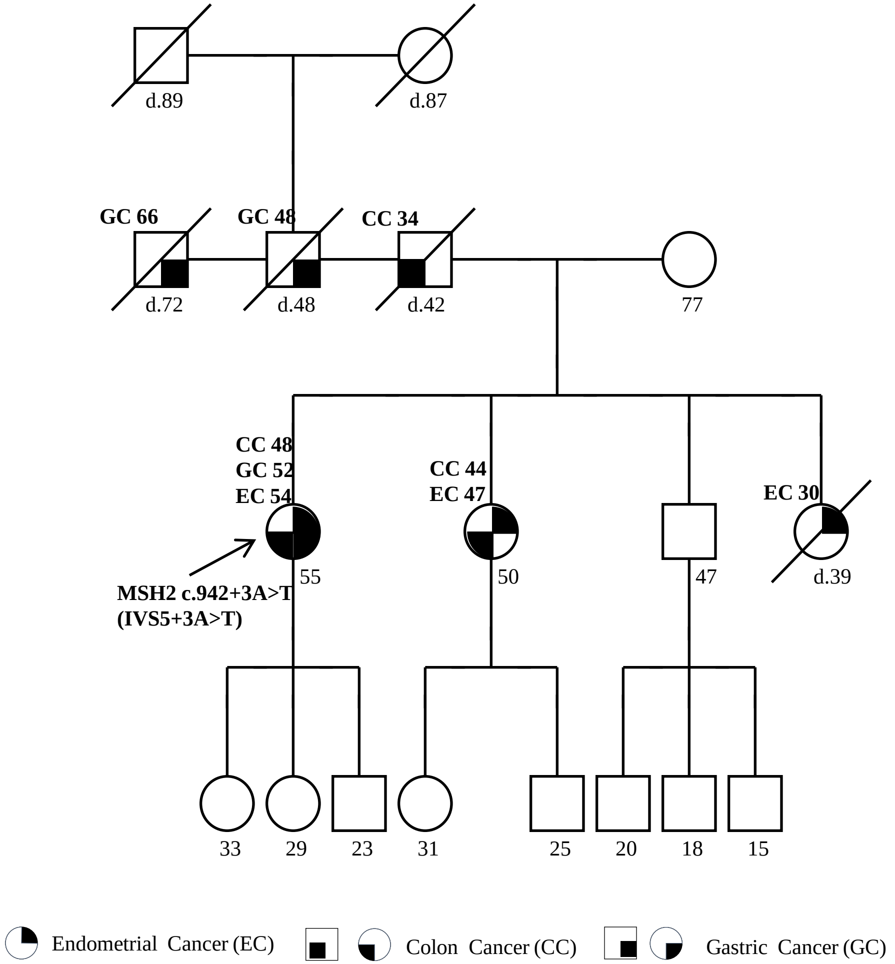
d.89
d.87
GC 66
GC 48
CC 34
d.72
d.48
d.42
77
CC 48
GC 52
EC 54
CC 44
EC 47
EC 30
55
50
47
d.39
MSH2 c.942+3A>T
(IVS5+3A>T)
33
29
23
31
25
20
18
15
Endometrial Cancer (EC)
Colon Cancer (CC)
Gastric Cancer (GC)

Family 7

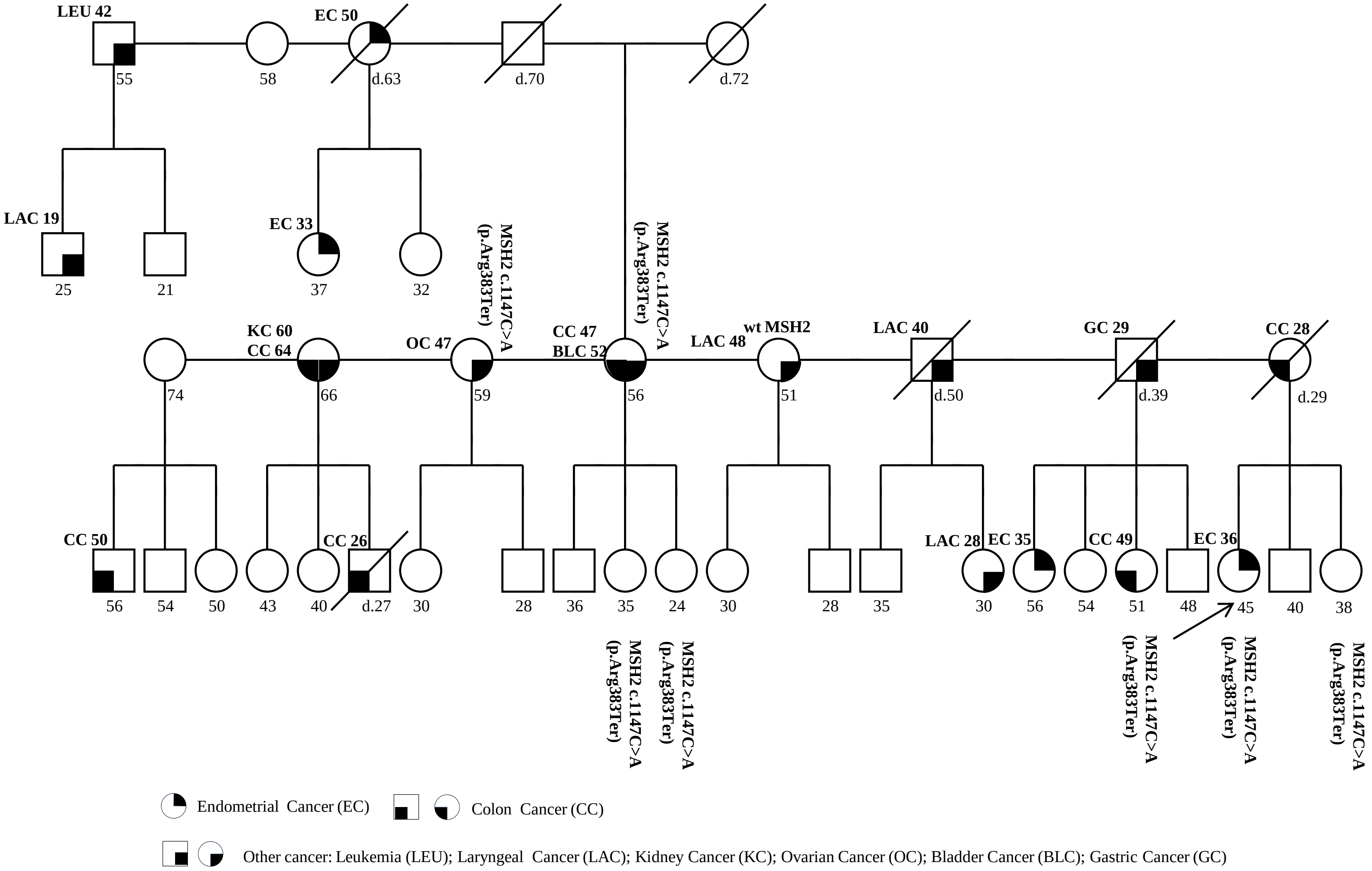
LEU 42
55
58
EC 50
d.63
d.70
d.72
LAC 19
25
21
EC 33
37
32
MSH2 c.1147C>A (p.Arg383Ter)
MSH2 c.1147C>A (p.Arg383Ter)
74
KC 60
CC 64
66
OC 47
59
CC 47
BLC 52
56
LAC 48
wt MSH2
51
LAC 40
d.50
GC 29
d.39
CC 28
d.29
CC 50
56
54
50
43
40
CC 26
d.27
30
28
36
35
24
30
28
35
LAC 28
30
EC 35
56
54
CC 49
51
48
EC 36
45
40
38
MSH2 c.1147C>A (p.Arg383Ter)
MSH2 c.1147C>A (p.Arg383Ter)
MSH2 c.1147C>A (p.Arg383Ter)
MSH2 c.1147C>A (p.Arg383Ter)
MSH2 c.1147C>A (p.Arg383Ter)
Endometrial Cancer (EC)
Colon Cancer (CC)
Other cancer: Leukemia (LEU); Laryngeal Cancer (LAC); Kidney Cancer (KC); Ovarian Cancer (OC); Bladder Cancer (BLC); Gastric Cancer (GC)

Family 8

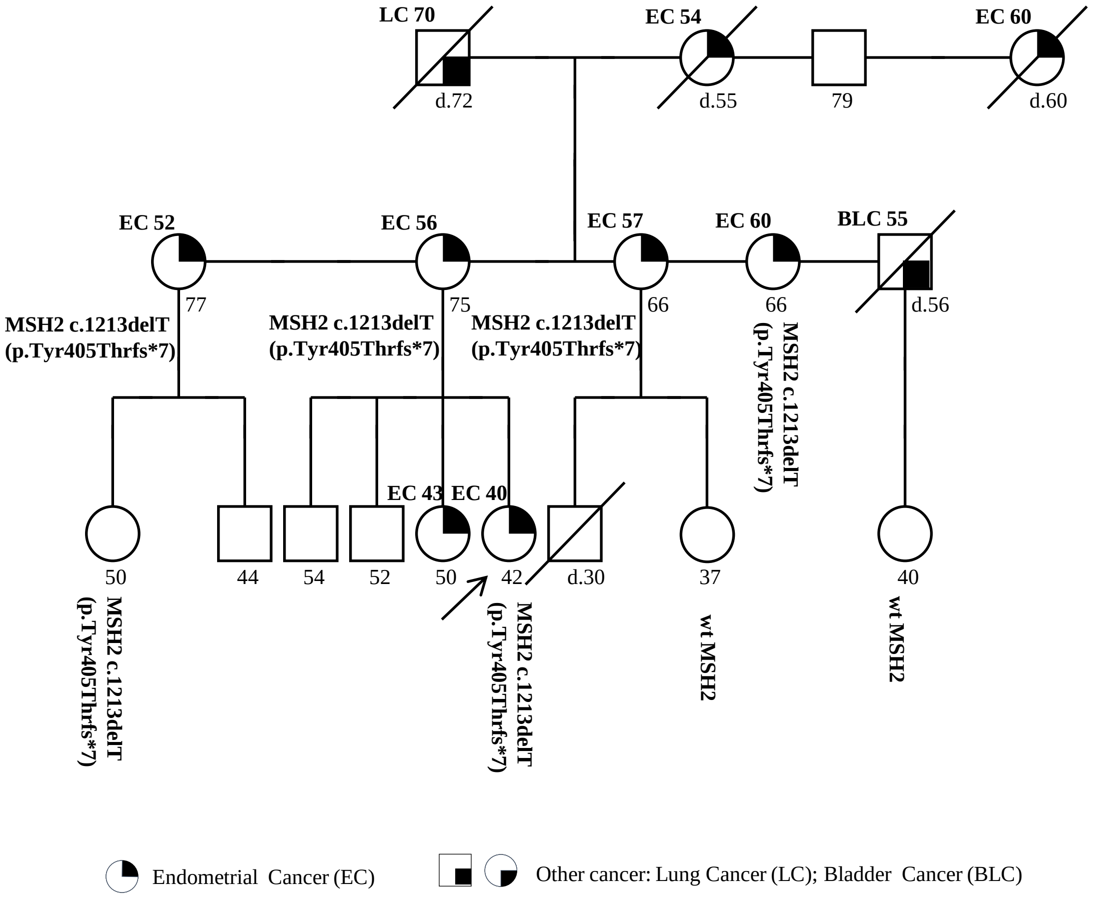
LC 70
d.72
EC 54
d.55
79
EC 60
d.60
EC 52
77
MSH2 c.1213delT
(p.Tyr405Thrfs*7)
EC 56
75
MSH2 c.1213delT
(p.Tyr405Thrfs*7)
EC 57
66
MSH2 c.1213delT
(p.Tyr405Thrfs*7)
EC 60
66
MSH2 c.1213delT
(p.Tyr405Thrfs*7)
BLC 55
d.56
50
MSH2 c.1213delT
(p.Tyr405Thrfs*7)
44
54
52
EC 43
50
EC 40
42
MSH2 c.1213delT
(p.Tyr405Thrfs*7)
d.30
37
wt MSH2
40
wt MSH2
Endometrial Cancer (EC)
Other cancer: Lung Cancer (LC); Bladder Cancer (BLC)

Family 9

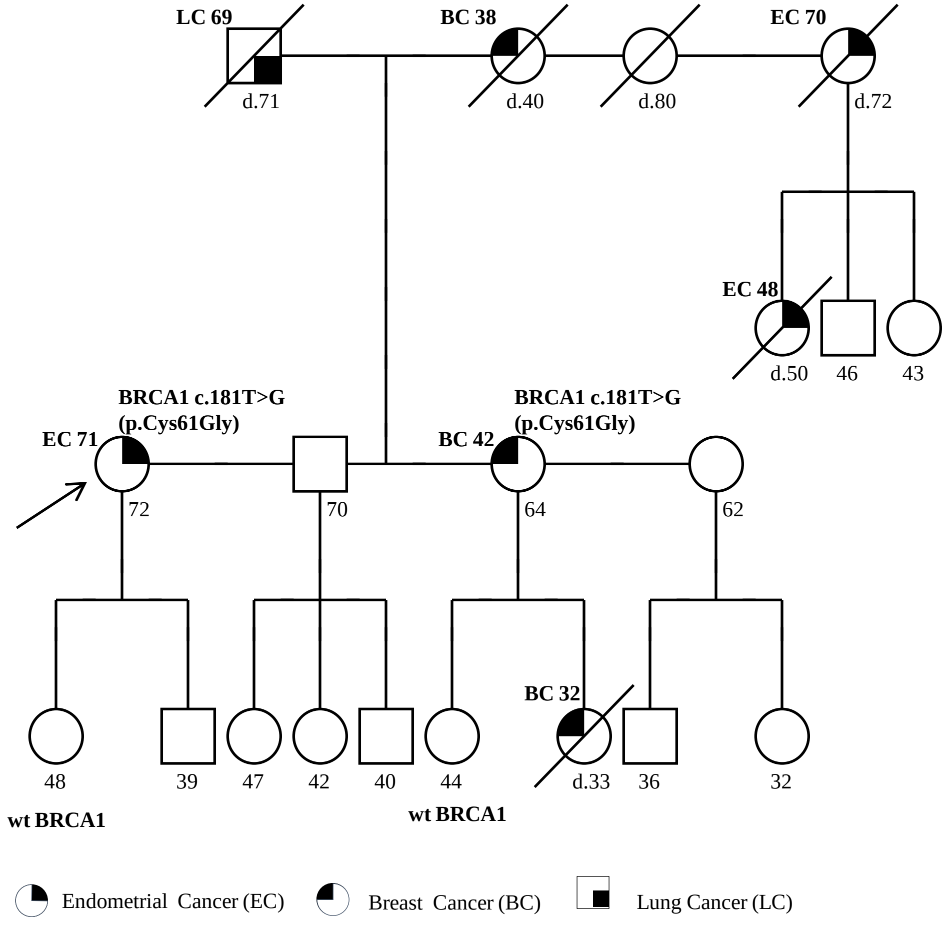

LC 69
d.71
BC 38
d.40
d.80
EC 70
d.72
EC 48
d.50
46
43
BRCA1 c.181T>G
(p.Cys61Gly)
EC 71
72
70
BRCA1 c.181T>G
(p.Cys61Gly)
BC 42
64
62
48
39
47
42
40
44
BC 32
d.33
36
32
wt BRCA1
wt BRCA1
Endometrial Cancer (EC)
Breast Cancer (BC)
Lung Cancer (LC)

Family 10

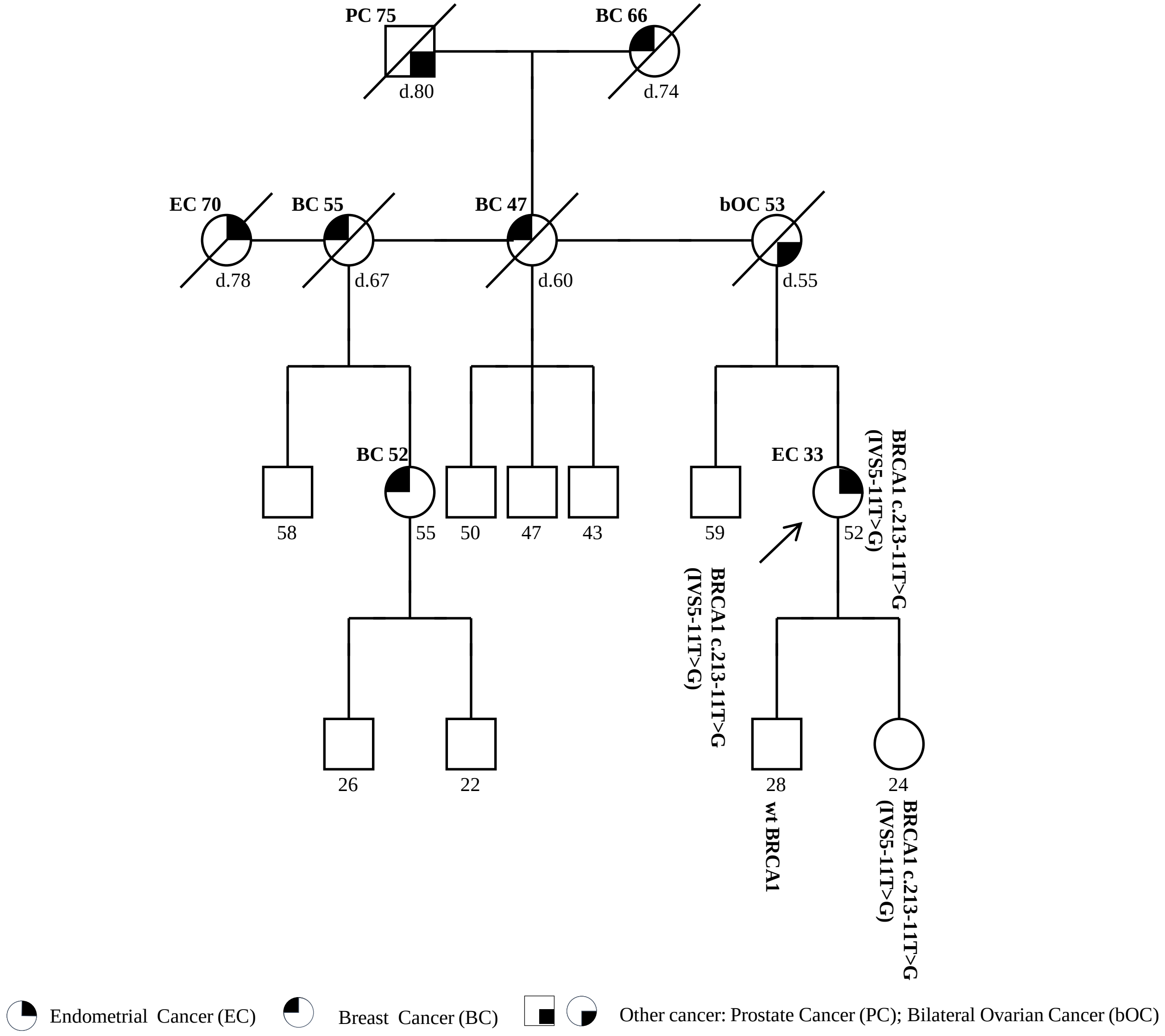

PC 75
d.80
BC 66
d.74
EC 70
d.78
BC 55
d.67
BC 47
d.60
bOC 53
d.55
BC 52
EC 33
58
55
50
47
43
59
52
BRCA1 c.213-11T>G (IVS5-11T>G)
BRCA1 c.213-11T>G (IVS5-11T>G)
26
22
28
24
wt BRCA1
BRCA1 c.213-11T>G (IVS5-11T>G)
Endometrial Cancer (EC)
Breast Cancer (BC)
Other cancer: Prostate Cancer (PC); Bilateral Ovarian Cancer (bOC)

Family 11

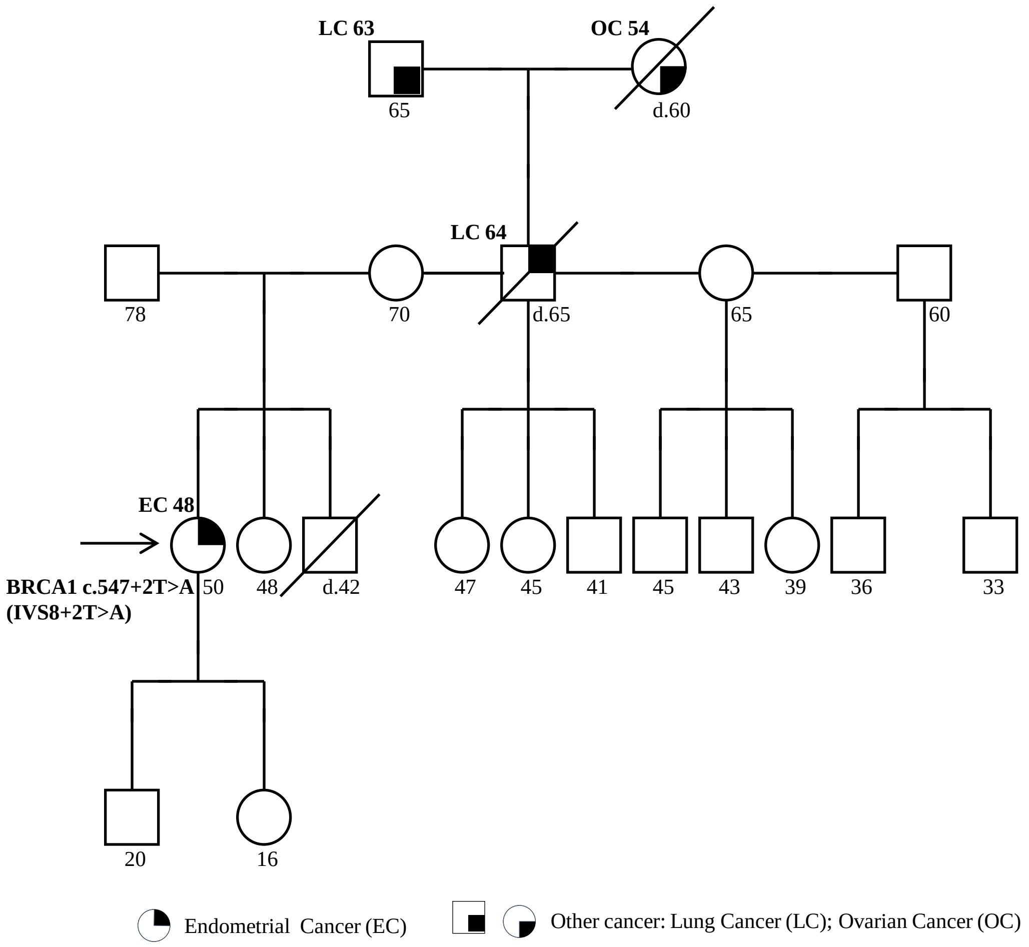
LC 63
OC 54
65
d.60
LC 64
78
70
d.65
65
60
EC 48
BRCA1 c.547+2T>A
(IVS8+2T>A)
50
48
d.42
47
45
41
45
43
39
36
33
20
16
Endometrial Cancer (EC)
Other cancer: Lung Cancer (LC); Ovarian Cancer (OC)

Family 12

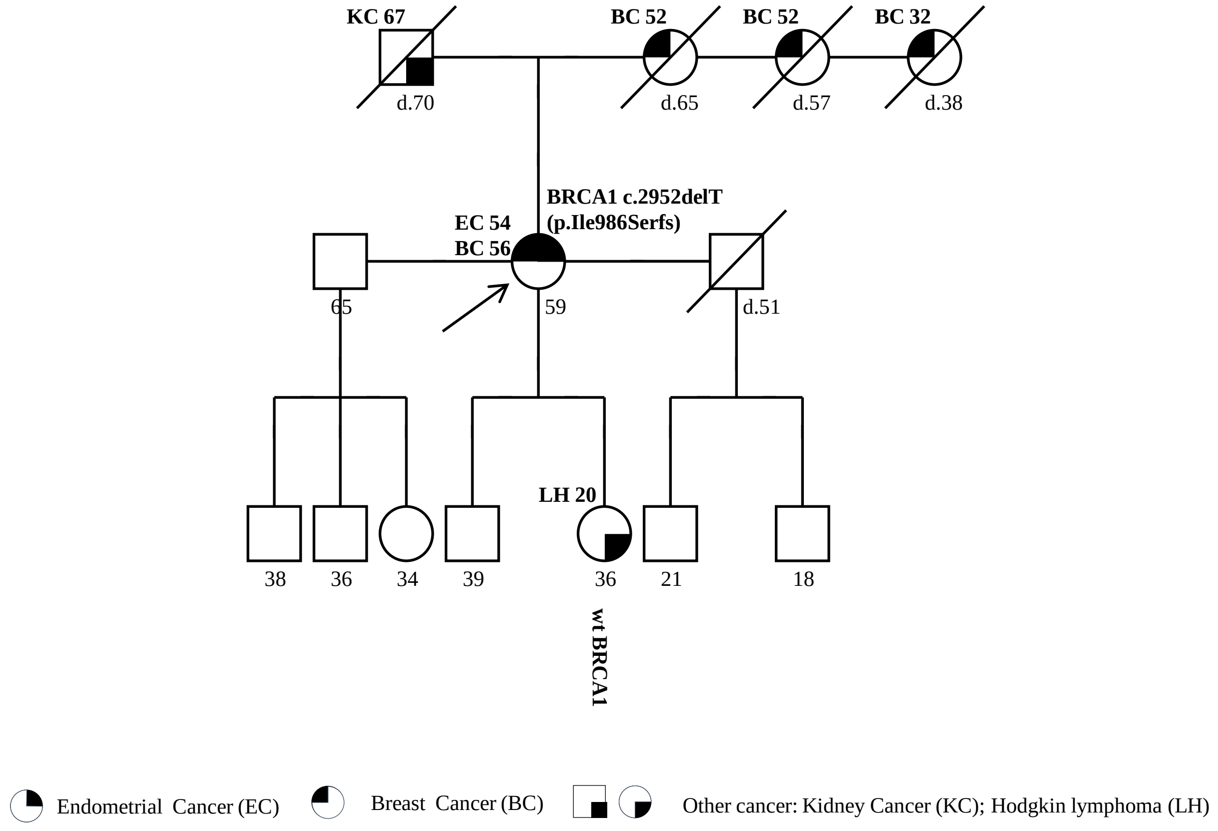
KC 67
d.70
BC 52
d.65
BC 52
d.57
BC 32
d.38
BRCA1 c.2952delT
(p.Ile986Serfs)
EC 54
BC 56
65
59
d.51
LH 20
38
36
34
39
36
21
18
wt BRCA1
Endometrial Cancer (EC)
Breast Cancer (BC)
Other cancer: Kidney Cancer (KC); Hodgkin lymphoma (LH)

Family 13

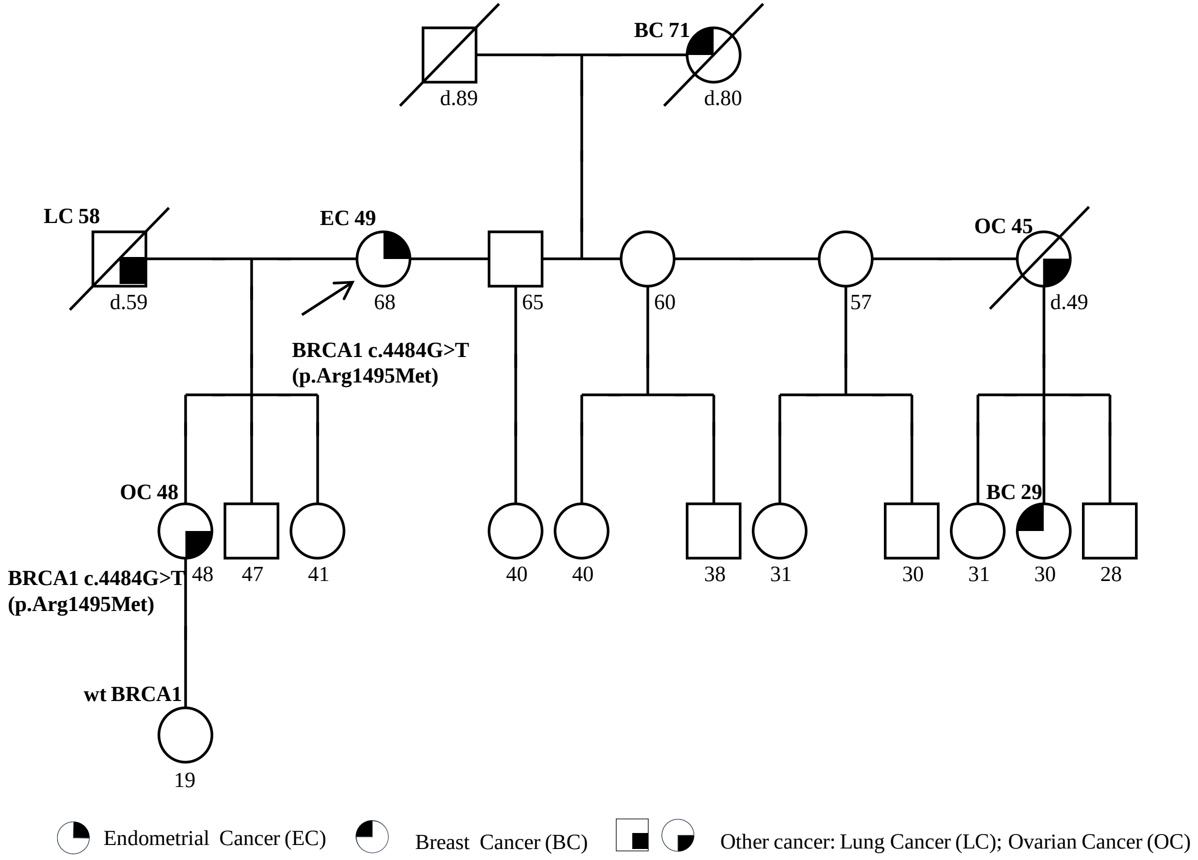

BC 71
d.89
d.80
LC 58
EC 49
OC 45
d.59
68
65
60
57
d.49
BRCA1 c.4484G>T
(p.Arg1495Met)
OC 48
BC 29
BRCA1 c.4484G>T
(p.Arg1495Met)
48
47
41
40
40
38
31
30
31
30
28
wt BRCA1
19
Endometrial Cancer (EC)
Breast Cancer (BC)
Other cancer: Lung Cancer (LC); Ovarian Cancer (OC)

Family 14

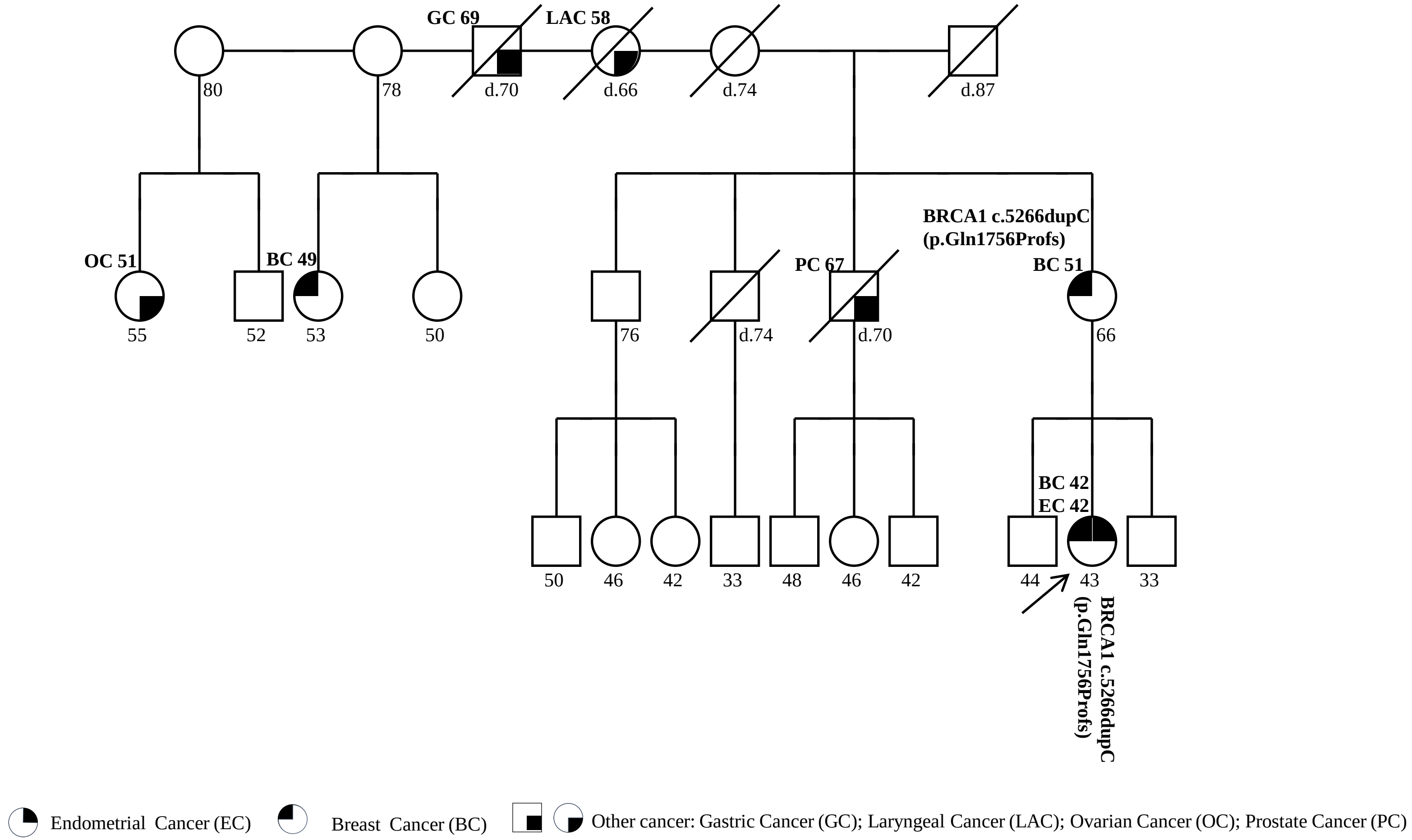
GC 69
LAC 58
80
78
d.70
d.66
d.74
d.87
BRCA1 c.5266dupC
(p.Gln1756Profs)
OC 51
BC 49
PC 67
BC 51
55
52
53
50
76
d.74
d.70
66
BC 42
EC 42
50
46
42
33
48
46
42
44
43
33
BRCA1 c.5266dupC
(p.Gln1756Profs)
Endometrial Cancer (EC)
Breast Cancer (BC)
Other cancer: Gastric Cancer (GC); Laryngeal Cancer (LAC); Ovarian Cancer (OC); Prostate Cancer (PC)

Family 15

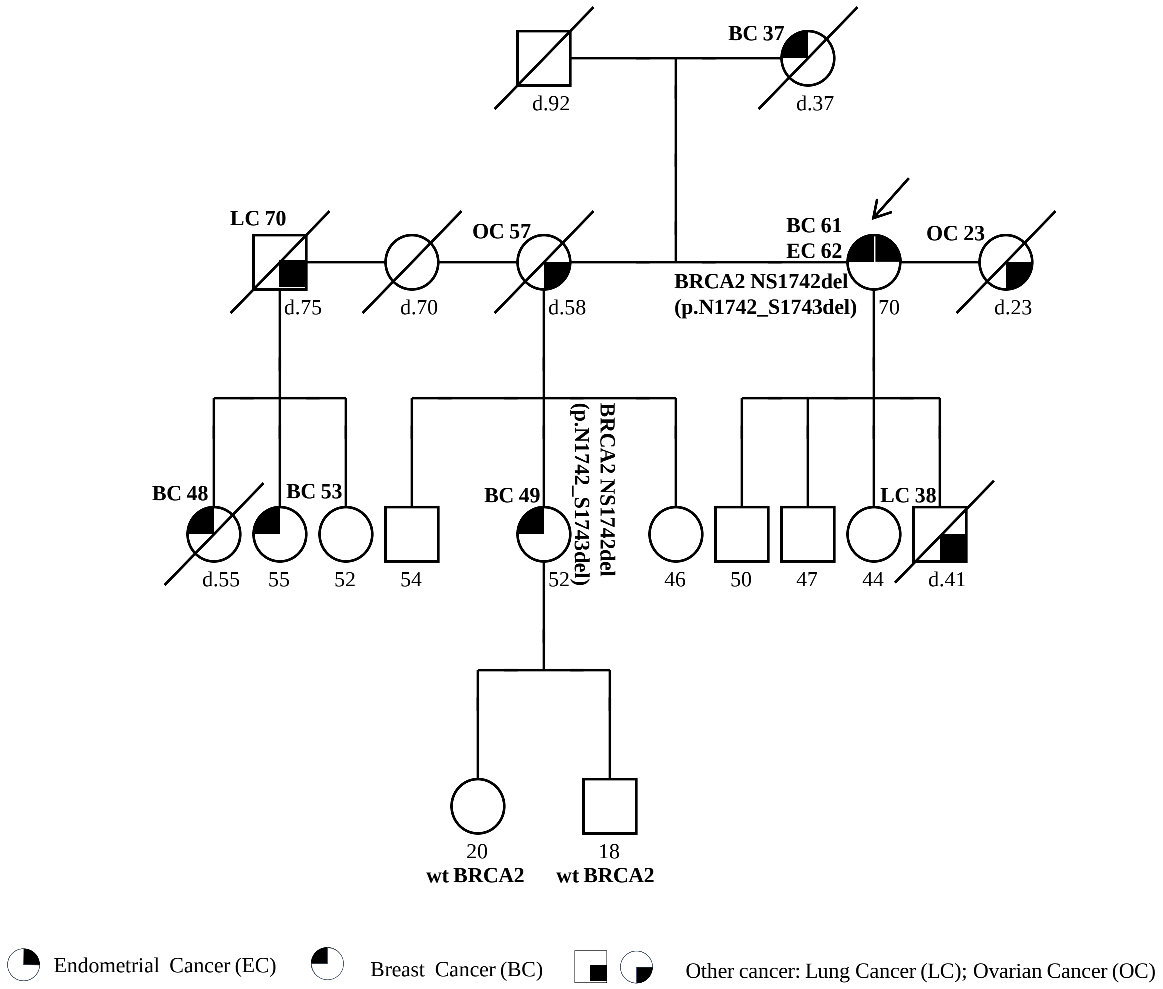
BC 37
d.92
d.37
LC 70
OC 57
BC 61
EC 62
OC 23
BRCA2 NS1742del
(p.N1742_S1743del)
d.75
d.70
d.58
70
d.23
BRCA2 NS1742del
(p.N1742_S1743del)
BC 48
BC 53
BC 49
LC 38
d.55
55
52
54
52
46
50
47
44
d.41
20
wt BRCA2
18
wt BRCA2
Endometrial Cancer (EC)
Breast Cancer (BC)
Other cancer: Lung Cancer (LC); Ovarian Cancer (OC)

Family 16

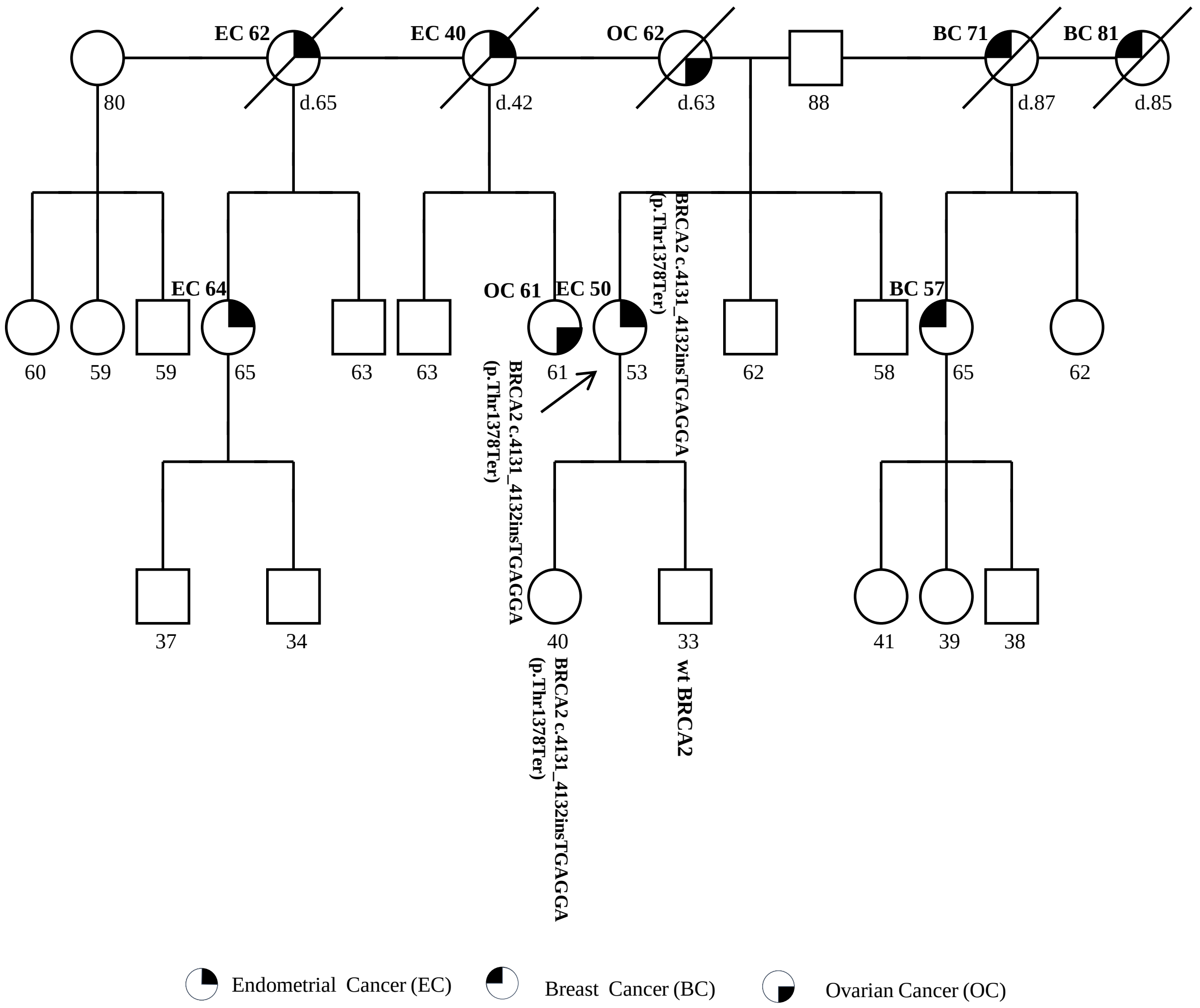

EC 62
EC 40
OC 62
BC 71
BC 81
80
d.65
d.42
d.63
88
d.87
d.85
EC 64
OC 61
EC 50
BC 57
60
59
59
65
63
63
61
53
62
58
65
62
BRCA2 c.4131_4132insTGAGGA (p.Thr1378Ter)
BRCA2 c.4131_4132insTGAGGA (p.Thr1378Ter)
37
34
40
33
41
39
38
BRCA2 c.4131_4132insTGAGGA (p.Thr1378Ter)
wt BRCA2
Endometrial Cancer (EC)
Breast Cancer (BC)
Ovarian Cancer (OC)

Family 17

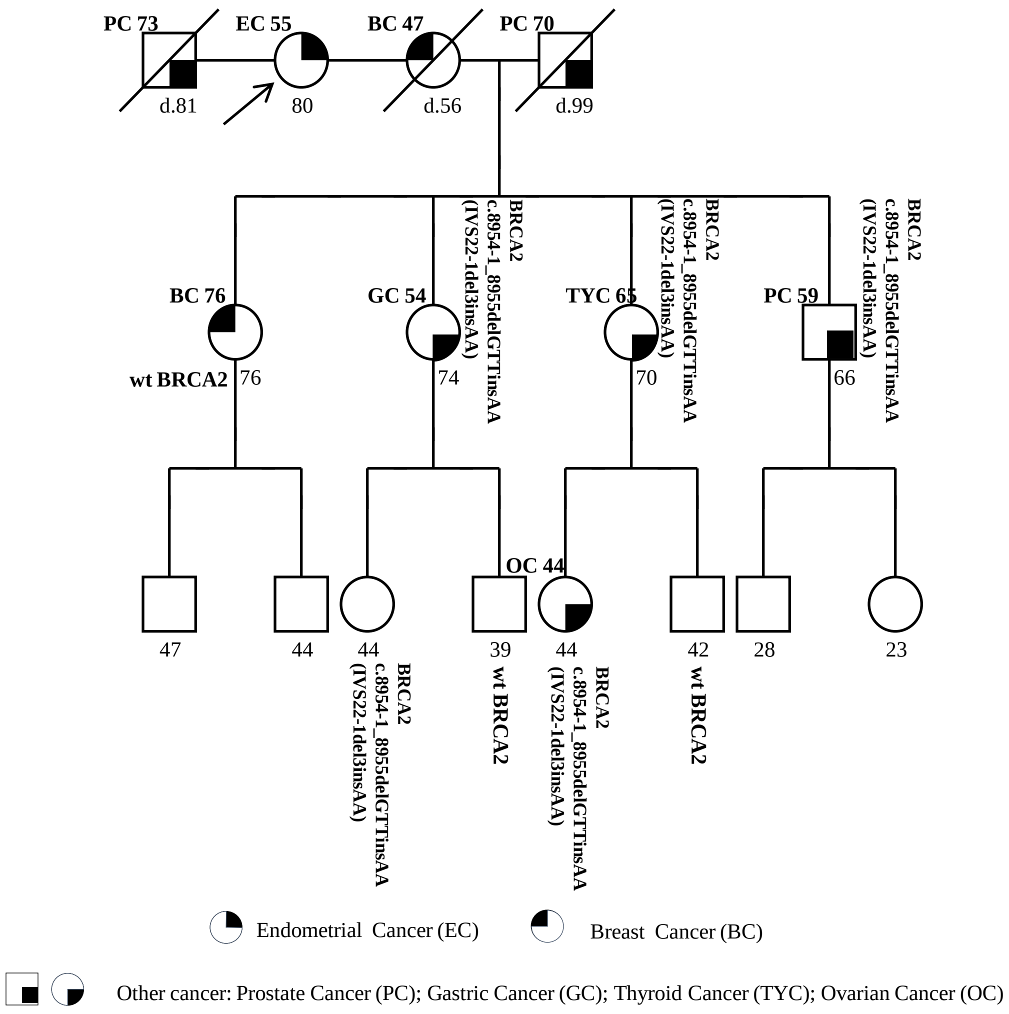
PC 73
EC 55
BC 47
PC 70
d.81
80
d.56
d.99
BC 76
GC 54
TYC 65
PC 59
wt BRCA2
76
74
70
66
BRCA2 c.8954-1_8955delGTTinsAA (IVS22-1del3insAA)
OC 44
47
44
44
39
44
42
28
23
wt BRCA2
Endometrial Cancer (EC)
Breast Cancer (BC)
Other cancer: Prostate Cancer (PC); Gastric Cancer (GC); Thyroid Cancer (TYC); Ovarian Cancer (OC)
