# Supplementary material for: Prevalence of mutations in BRCA and MMR genes in patients affected with hereditary endometrial cancer
Source: Med Oncol. 2021 Jan 23;38(2):13. doi: 10.1007/s12032-021-01454-5 (PMC7826304; doi:10.1007/s12032-021-01454-5)
Supplement: Supplementary file 2 — Supplementary information 2 (DOCX 19 kb) [file 12032_2021_1454_MOESM2_ESM.docx]

Supplementary Figure Legends

Fig. S1 Pedigree of family 1 with LS. The ages at diagnosis are indicated in brackets

Fig. S2 Pedigree of family 2 with LS. The ages at diagnosis are indicated in brackets

Fig. S3 Pedigree of family 3 with LS. The ages at diagnosis are indicated in brackets

Fig. S4 Pedigree of family 4 with LS. The ages at diagnosis are indicated in brackets

Fig. S5 Pedigree of family 5 with LS. The ages at diagnosis are indicated in brackets

Fig. S6 Pedigree of family 6 with LS. The ages at diagnosis are indicated in brackets

Fig. S7 Pedigree of family 7 with LS. The ages at diagnosis are indicated in brackets

Fig. S8 Pedigree of family 8 with LS. The ages at diagnosis are indicated in brackets

Fig. S9 Pedigree of family 9 with HBOC. The ages at diagnosis are indicated in brackets

Fig. S10 Pedigree of family 10 with HBOC. The ages at diagnosis are indicated in brackets

Fig. S11 Pedigree of family 11 with HBOC. The ages at diagnosis are indicated in brackets

Fig. S12 Pedigree of family 12 with HBOC. The ages at diagnosis are indicated in brackets

Fig. S13 Pedigree of family 13 with HBOC. The ages at diagnosis are indicated in brackets

Fig. S14 Pedigree of family 14 with HBOC. The ages at diagnosis are indicated in brackets

Fig. S15 Pedigree of family 15 with HBOC. The ages at diagnosis are indicated in brackets

Fig. S16 Pedigree of family 16 with HBOC. The ages at diagnosis are indicated in brackets

Fig. S17 Pedigree of family 17 with HBOC. The ages at diagnosis are indicated in brackets
